# Stoichiometry of the Gene Products From the Tetrachloroethene Reductive Dehalogenase Operon *pceABCT*

**DOI:** 10.3389/fmicb.2022.838026

**Published:** 2022-02-23

**Authors:** Lorenzo Cimmino, Adrien W. Schmid, Christof Holliger, Julien Maillard

**Affiliations:** ^1^Laboratory for Environmental Biotechnology, Institute for Environmental Engineering, Ecole Polytechnique Fédérale de Lausanne, Lausanne, Switzerland; ^2^Protein Core Facility, Ecole Polytechnique Fédérale de Lausanne, Lausanne, Switzerland

**Keywords:** organohalogens, anaerobic respiration, Firmicutes (Bacillota), gene product stoichiometry, *rdh* gene clusters, operon, PRM quantitative proteomics

## Abstract

Organohalide respiration (OHR) is a bacterial anaerobic process that uses halogenated compounds, e.g., tetrachloroethene (PCE), as terminal electron acceptors. Our model organisms are *Dehalobacter restrictus* strain PER-K23, an obligate OHR bacterium (OHRB), and *Desulfitobacterium hafniense* strain TCE1, a bacterium with a versatile metabolism. The key enzyme is the PCE reductive dehalogenase (PceA) that is encoded in the highly conserved gene cluster (*pceABCT*) in both above-mentioned strains, and in other Firmicutes OHRB. To date, the functions of PceA and PceT, a dedicated molecular chaperone for the maturation of PceA, are well defined. However, the role of PceB and PceC are still not elucidated. We present a multilevel study aiming at deciphering the stoichiometry of *pceABCT* individual gene products. The investigation was assessed at RNA level by reverse transcription and (quantitative) polymerase chain reaction, while at protein level, proteomic analyses based on parallel reaction monitoring were performed to quantify the Pce proteins in cell-free extracts as well as in soluble and membrane fractions of both strains using heavy-labeled reference peptides. At RNA level, our results confirmed the co-transcription of all *pce* genes, while the quantitative analysis revealed a relative stoichiometry of the gene transcripts of *pceA, pceB, pceC*, and *pceT* at ~ 1.0:3.0:0.1:0.1 in *D. restrictus*. This trend was not observed in *D. hafniense* strain TCE1, where no substantial difference was measured for the four genes. At proteomic level, an apparent 2:1 stoichiometry of PceA and PceB was obtained in the membrane fraction, and a low abundance of PceC in comparison to the other two proteins. In the soluble fraction, a 1:1 stoichiometry of PceA and PceT was identified. In summary, we show that the *pce* gene cluster is transcribed as an operon with, however, a level of transcription that differs for individual genes, an observation that could be explained by post-transcriptional events. Despite challenges in the quantification of integral membrane proteins such as PceB and PceC, the similar abundance of PceA and PceB invites to consider them as forming a membrane-bound PceA_2_B protein complex, which, in contrast to the proposed model, seems to be devoid of PceC.

## 1. Introduction

Members of the genera *Dehalobacter* and *Desulfitobacterium* strongly differ in their overall metabolic strategies, despite a phylogenetically close relationship (Futagami and Furukawa, 2016; Maillard and Holliger, 2016), as they both belong to the Firmicutes (newly named as Bacillota Oren and Garrity, 2021). *Dehalobacter* sp. is considered as an obligate organohalide-respiring bacterium (OHRB), while *Desulfitobacterium* spp. are metabolically versatile with the ability to use fumarate, nitrate, sulfite, thiosulfate, ferrous iron and many more compounds as electron acceptors besides a few organohalogens. In addition, most isolates of *Desulfitobacterium* spp. have been also reported to grow fermentatively on pyruvate (Kruse et al., 2017). On the one hand, *D. restrictus* strain PER-K23 has been isolated from a tetrachloroethene (PCE) dechlorinating enrichment culture and reported to grow exclusively *via* organohalide respiration (OHR) with acetic acid and CO_2_ as carbon sources, H_2_ as electron donor, tetrachloroethene (PCE) or trichloroethene (TCE) as sole electron acceptors (Holliger et al., 1998). On the other hand, *D. hafniense* strain TCE1 has been isolated from a chloroethene-contaminated aquifer and was reported to dechlorinate PCE and TCE (Gerritse et al., 1999).

The sequenced genomes of *Desulfitobacterium* spp. and *D. restrictus* are characterized by the presence of multiple reductive dehalogenase homologous (*rdh*) gene clusters, including *rdhA* which encodes the key catalytic enzyme for OHR. *D. restrictus* strain PER-K23 displays twenty-five different *rdhA* genes (Kruse et al., 2013), while OHR members of the *Desulfitobacterium* genus harbor between one and seven *rdhA* genes in their genome (Kruse et al., 2017). Strikingly, both *D. restrictus* strain PER-K23 and *D. hafniense* strains TCE1 share the highly conserved (99% amino acid sequence identity) and well-characterized PCE reductive dehalogenase, which is encoded by the *pceA* gene (Maillard et al., 2003) and is part of a four-gene cluster (*pceABCT*) (Supplementary Figure 1A), that also displays 99% DNA sequence identity between the two organisms (Maillard et al., 2005). The conserved *pceABCT* gene cluster, and more generally, *rdhABCT* gene clusters, have been found in the genera *Dehalobacter and Desulfitobacterium* exclusively (Duret et al., 2012). A variant of this gene cluster that displays a different gene order and duplicated genes, and that shares a low sequence homology with *rdhABCT*, was identified on a genomic island in the chromosome of *Geobacter lovleyi* strain SZ (Wagner et al., 2012), and was, therefore, not considered as part of the conserved *rdhABCT* gene clusters. At genomic level, the *pce* gene cluster in *D. hafniense* strain TCE1 is located on the active composite transposon *Tn*-Dha1 (Maillard et al., 2005; Duret et al., 2012), a structure that has been found with some variations in other *Desulfitobacterium* spp. (Futagami et al., 2006; Duret et al., 2012; Goris et al., 2015). In *D. hafniense* strain TCE1, the presence of the transposon flanking the *pceABCT* gene cluster resulted in the constitutive expression of the *pce* genes, while a gradual loss of the entire *pce* gene cluster was confirmed during serial sub-cultivation of strain TCE1 under growth conditions devoid of PCE (Duret et al., 2012). By contrast, no transposon structure is found around the *pceABCT* gene cluster of *D. restrictus*.

Respiratory reductive dehalogenase genes are typically organized in gene clusters composed of *rdhA*, the gene encoding the catalytic enzyme, and at minimum *rdhB*, coding for the putative membrane anchor protein that attaches RdhA at the cytoplasmic membrane. The *rdhAB* or *rdhBA* genes represent the minimal *rdh* gene sets so far detected (Hug et al., 2013), with the exception of a few strains of *Dehalogenimonas* spp. that display some isolated *rdhA* genes (Siddaramappa et al., 2012). The operon nature of *rdhAB* or *rdhBA* genes has been revealed for several gene clusters (Neumann et al., 1998; Smidt et al., 2000; Maillard et al., 2005; Tsukagoshi et al., 2006). A robust reductive dehalogenase activity associated with the membrane fraction of selected OHRB (Ni et al., 1995; Schumacher and Holliger, 1996; Maillard et al., 2003) corroborate the hypothesis that respiratory RdhA enzymes are bound to the cytoplasmic membrane, although they do not display any transmembrane helix in the maturated form, as demonstrated by the crystal structure of the dimeric PceA in *Sulfurospirillum multivorans* (Bommer et al., 2014). The genetic vicinity of *rdhB*, the sequence of which was predicted to form a short integral membrane protein of ~ 100 amino acids with two or three transmembrane helices (Schubert et al., 2018), invites considering the RdhB protein as the membrane anchor of their cognate RdhA. However, so far only indirect evidence has been obtained for their interaction (Seidel et al., 2018). Additional accessory proteins are often encoded adjacently to *rdhAB* or *rdhBA* operons (for reviews, see Kruse et al., 2017; Maillard and Willemin, 2019). A well-studied example is the gene cluster encoding the chlorophenol reductive dehalogenase in *D. dehalogenans*, namely *cprTKZEBACD*, which harbors six additional genes in the direct vicinity of the *cprBA* operon (Smidt et al., 2000).

For the two accessory genes present in the *pceABCT* gene cluster, a function has only been clearly established for the gene product of *pceT*. PceT and other RdhT proteins are molecular chaperones most likely assisting in the correct folding of the catalytic subunit. It has been shown to bind to the Twin-arginine translocation (Tat) signal sequence of PceA (Morita et al., 2009; Maillard et al., 2011). Moreover, in proteomic analyses conducted on *D. hafniense* strain TCE1 (Prat et al., 2011) and strain Y51 (Reinhold et al., 2012), or on *D. restrictus* strain PER-K23 (Rupakula et al., 2013), PceT has been clearly detected. PceC (and more generally RdhC proteins) encodes a predicted integral membrane protein (with six transmembrane helices) harboring a flavin mononucleotide (FMN)-binding domain and two conserved CX_3_CP amino acid motifs. Initially, based on sequence similarity, RdhC was proposed to play a similar function as NosR- and NirI-type regulatory proteins (Smidt et al., 2000; Maillard et al., 2005). Experimental work has confirmed the presence of a covalently-bound FMN cofactor in the protein domain of PceC that faces the outside of the cytoplasmic membrane (Buttet et al., 2018). These data suggested that RdhC proteins could play a role in electron transfer and form a membrane-bound protein complex with RdhA and RdhB (Maillard and Holliger, 2016), although there is no experimental evidence to support this hypothesis.

In the present study, OHRB harboring diverse metabolic strategies, i.e., the obligate *D. restrictus* strain PER-K23 and the versatile *Desulfitobacterium hafniense* strain TCE1, were investigated to elucidate the stoichiometry of the *pceABCT* individual gene products at RNA and protein levels.

## 2. Materials and Methods

### 2.1. Bacterial Strains and Growth Conditions

*Dehalobacter restrictus* strain PER-K23 (DSM 9455) and *Desulfitobacterium hafniense* strain TCE1 (DSM 12704) were cultivated anaerobically at 30°C under agitation (100 rpm). Volumes of 40 and 200 mL (in 100 and 500-mL serum flasks, respectively) of medium were prepared where the head space was replaced by a mixture of N_2_/CO_2_ (80/20%) as described earlier (Comensoli et al., 2017) with the modifications that cyanocobalamin was supplemented at 50 μM (final concentration) and that dicyanocobinamide was used instead of cyanocobalamin for the cultivation of *D. restrictus* strain PER-K23. Dicyanocobinamide was used here to allow *D. restrictus* to decorate the corrinoid cofactor with the lower ligand of its choice, likely with purine as suggested earlier (Wang et al., 2017), and in studies on *Desulfitobacterium* (Yan et al., 2018; Schubert et al., 2019). Completed medium was supplemented with acetic acid (5 mM) as carbon source, the head space replaced by a gas mixture of H_2_/CO_2_ (80/20%) to provide H_2_ as electron donor, and inoculated with 5% (v/v) of a preculture. To study OHR metabolism, cultures were supplemented with PCE as electron acceptor (nominal concentration of 10 mM) in a biphasic system. For *D. hafniense* strain TCE1 1% (v/v) of a 2 M PCE stock solution in hexadecane was used (with an estimated aqueous concentration of 0.4 mM), while for *D. restrictus* it was 4% (v/v) of a 500 mM PCE stock solution, thus keeping a lower aqueous PCE concentration (0.1 mM). Indeed, we have observed that a high PCE concentration had a slight toxic effect on the growth of *D. restrictus*. *D. hafniense* strain TCE1 was alternatively cultivated with 40 mM of pyruvate as sole carbon and energy source. The cultures were routinely transferred to fresh medium after 10 days of cultivation.

### 2.2. Spike Experiment

*D. hafniense* strain TCE1 was cultivated in anaerobic flasks containing 40-mL of medium with 40 mM sodium pyruvate as carbon and energy source (thus replacing acetic acid, H_2_ and PCE). This culture set-up was used for an experiment where PCE was spiked in after 24 h of growth on pyruvate at an optical density at 600 nm (OD600) of 0.08. Upon PCE addition, four replicate culture flasks were incubated for 5 h before biomass collection. The experiment conducted on *D. restrictus* cells growing with H_2_ and PCE was performed slightly differently as no spike nor alternative growth conditions are possible. There, the transcription of the *pce* genes was compared to the corresponding DNA gene copy number from sample aliquots obtained during the RNA extraction procedure. For harvesting, the cultures were transferred to 50-mL Falcon tubes, centrifuged for 5 min at 4000 × *g* and the biomass pellets were quickly resuspended in 0.5 mL of RNAprotect Bacteria Reagent (Qiagen AG, Hombrechtikon, Switzerland) and transferred to 1.5-mL Eppendorf tubes. After 3 min of incubation at room temperature, the biomass was pelleted again by 3 min of centrifugation at 10,000 × *g*, and stored at −80°C until use.

### 2.3. RNA Extraction

Biomass pellets resulting from 40-mL cultures were resuspended by pipetting in 0.5 mL of TRIzol reagent (Thermo Fisher Scientific SARL, Ecublens, Switzerland) and incubated for 5 min at room temperature. A volume of 0.1 mL of chloroform was added and the mixture was vortexed vigorously for 15 s, incubated for 2 min and centrifuged at 10,000 × *g* for 15 min. The supernatant was carefully collected, mixed with an equal volume of 100% ethanol and purified using the Direct-zol RNA MiniPrep kit (Zymo Research, Lucerna-Chem AG, Luzern, Switzerland), following the manufacturer's instructions with the following modifications. The nucleic acids were eluted in 50 μL of water and a 5-μL aliquot was withdrawn to be used as DNA reference sample in quantitative PCR. The remaining elution was then supplemented with 5 μL of DNase buffer and 1 μL of DNase I enzyme (DNase Max Kit, Qiagen), and incubated for 30 min at 37°C. The DNase I enzyme was then removed with 5 μL of DNase Removal Resin according to the instructions. RNA samples were quantified using the Qubit RNA HS Assay Kit (Thermo Fisher Scientific) and stored at −80°C until further use.

### 2.4. Reverse Transcription, PCR and Quantitative PCR

Depending on the RNA samples, between 250 and 1,000 ng of RNA were transcribed to complementary DNA (cDNA) using the GoScriptTM Reverse Transcriptase Kit with random primers (Promega AG, Dübendorf, Switzerland) following the manufacturer's instructions. The resulting cDNA was diluted 10 × with ddH_2_O and either subjected to PCR or quantitative PCR (qPCR). A typical mixture for PCR in 20 μL contained the following: 4 μL of MyTaq Reaction Buffer, 1 μL of each of the 10 μM primers (Supplementary Table 1), 0.4 μL of MyTaq DNA Polymerase (Meridian Bioscience, LABGENE Scientific SA, Châtel-St-Denis, Switzerland), and 1 μL of cDNA template. The PCR program was: 1 min at 95°C, then 30 cycles of 15 s at 95°C, 15 s at 52°C, and 10 s at 72°C, followed by 5 min at 72°C. PCR products were visualized on 2% agarose gels following standard procedures. For qPCR, technical duplicates were run from three to four biological replicates of each strain and each growth condition considered. Ten μL reactions were prepared on the Myra Pipetting Robot (Bio Molecular Systems, LABGENE Scientific SA) with the following composition: 5 μL of SensiFAST SYBR No-ROX reagent (Meridian Bioscience), 0.2 μL of each primer at 10 μM, 2.1 μL of ddH_2_O and 2.5 μL of cDNA template. The qPCR was run on a MIC Real-Time PCR System (Bio Molecular Systems) with the following program: 2 min at 95°C, 40 cycles of 5 s at 95°C, 15 s at 60–62°C (depending on the target gene), 20 s at 72°C, followed by a 4 min denaturation ramp from 72 to 95°C. Data analysis was performed with the in-built MIC software using the relative quantification REST mode and *rpoB* as reference gene. In case of spike experiments, the samples obtained from the non-spiked cultures (with pyruvate) were used as control. Raw data, qPCR melting curves and statistical analysis output are given in Supplementary Table 1.

### 2.5. Cell Harvest, Fractionation and Protein Extraction

For proteomic analysis, *D. restrictus* and *D. hafniense* strain TCE1 were cultivated anaerobically in duplicates as described above. The growth was monitored at OD600 and the biomass was collected after three days of incubation corresponding to exponential growth. The biomass pellets were washed three times in 50 mM Tris-HCl (pH 7.5), and stored at −80°C until use. The biomass pellets were resuspended in 50 mM Tris-HCl buffer supplemented with the complete EDTA-free protease inhibitor cocktail (Merck, Zug, Switzerland) and with a few crystals of DNase I (Merck) at a ratio of 5 mL per g of cells (wet weight). The cells were lysed with five rounds of sonication at 60% amplitude on the Sonic Dismembrator FB120 (Fisher Scientific, Reinach, Switzerland). After 15 min of a mild centrifugation (500 × *g*, 4°C), the cell-free extract (CFE) was obtained from the supernatant and the pellet of unbroken cells was discarded. Soluble and membrane fractions were obtained by ultracentrifugation (90,000 × *g*, 4°C and 30 min) of the cell-free extract. The top half of the resulting supernatant was transferred to a new tube and subjected to an additional ultracentrifugation wherefrom the top 80% of the supernatant was collected and represented the soluble fraction (SF) sample. On the other hand, the pellet obtained from the first ultracentrifugation step was resuspended in 50 mM Tris-HCl buffer containing the protease inhibitor and transferred in a new Eppendorf tube. Ultracentrifugation was applied again and the resulting membrane pellet was resuspended with 50 mM Tris-HCl buffer supplemented with the protease inhibitor cocktail to obtain the membrane faction (MF). The total protein content was measured for CFE, SF, and MF samples with the Pierce BCA kit assay (Thermo Scientific, Basel, Switzerland) and technical replicates of 10 μg of proteins were aliquoted in new tubes, supplemented with 1% RapiGest SF Surfactant (Waters AG, Baden-Dättwil, Switzerland) and stored at 4°C until use.

### 2.6. In-solution Sample Digestion

Cell-free extract, soluble and membrane samples were reduced and alkylated as outlined below followed by in-solution overnight digestion at 37°C with Trypsin/LysC proteases (Thermo Fisher Scientific). RapiGest-treated samples were generally prepared according the manufacturer's instructions. Protein digests were then subjected to C18 stage tip cleaning, dried in a speed-vacuum and stored at −20°C.

### 2.7. Discovery LC-MS/MS Analysis

Shotgun mass spectrometry (MS) analysis was performed on an Orbitrap Exploris 480 mass spectrometer (Thermo Fisher Scientific) coupled to a nano-UPLC Dionex pump. For liquid chromatography (LC)-MS/MS analysis, Trypsin/LysC digested samples were resuspended in 30-60 μL of a mobile phase [solvent A: 2% acetonitrile (ACN) in water, 0.1% formic acic (FA)] and then separated by reversed-phase chromatography using a Dionex Ultimate 3000 RSLC nanoUPLC system on a home-made 75 μm ID × 50 cm C18 capillary column (Reprosil-Pur AQ 120 Å, 1.9 μm) in-line connected with the MS instrument. Peptides were separated by applying a non-linear 150 min gradient ranging from 99% solvent A to 90% solvent B (90% ACN and 0.1% FA) at a flow rate of 250 nl/min. For spectral library and charge state determination of the peptides from PceA, PceB, PceC, PceT and the F1 α-subunit of the ATP synthase, the MS instrument was operated in data-dependent mode (DDA). Full-scan MS spectra (300-1500 m/z) were acquired at a resolution of 120,000 at 200 m/z. Data-dependent MS/MS spectra were recorded followed by HCD (higher-energy collision dissociation) fragmentation on the ten most intense signals per cycle (2 s), using an isolation window of 1.4 m/z. HCD spectra were acquired at a resolution of 60,000 using a normalized collision energy of 32 and a maximum injection time of 100 ms. The automatic gain control (AGC) was set to 100,000 ions. Charge state screening was enabled such that unassigned and charge states higher than six were rejected. Precursors intensity threshold was set at 5,000. Precursor masses previously selected for MS/MS measurement were excluded from further selection for a duration of 20 s, and the mass exclusion window was set at 10 ppm.

The mass spectrometry proteomics discovery data have been deposited to the ProteomeXchange Consortium *via* the PRIDE partner repository (Perez-Riverol et al., 2019), with the dataset identifiers PXD030941 and 10.6019/PXD030941.

### 2.8. Selection of Signature Peptides for Parallel Reaction Monitoring Proteomics

For parallel reaction monitoring (PRM)-based quantitative proteomics, alongside with the *pceABCT* encoded proteins, the F1 α-subunit of the ATP synthase was included as housekeeping protein, since it was detected with a high sequence coverage and reproducible signal intensity in a preliminary MS analysis performed on cell-free extracts of *D. restrictus* (data not shown) and in the discovery MS analysis (Supplementary Figure 2). The selection of signature peptides was based on unique proteotypic peptide sequences and features that enhance chemical stability. Priority was given to those peptides that were previously identified in the discovery dataset with high MS/MS spectral quality. Peptides containing cysteine or methionine residues were excluded. Peptide uniqueness was confirmed by searching against the *D. restrictus* proteome database. An additional criterion was that the selected peptides display a fully conserved amino acid sequence in the protein homologues from both *D. restrictus* and *D. hafniense* strain TCE1. Synthetic, accurately quantified heavy-isotope labeled reference peptides, with either C-terminal heavy lysine (K) or arginine (R) for PceA, PceB, PceC, PceT and the α-F1 subunit of ATP synthase were purchased from JPT Peptide Technologies GmbH (Berlin, Germany).

### 2.9. PRM-Based Quantitative Proteomics

PRM-based proteomics was conducted on biological duplicates of samples from *D. restrictus* and *D. hafniense* strain TCE1. Each sample (CFE, SF, MF) was analyzed as technical triplicates. PRM analysis was performed using a Q-Exactive hybrid quadrupole-orbitrap mass spectrometer (Q-OT, Thermo Scientific). The MS/MS resolution was set at 17,500 (at m/z 200). The maximum fill time was set at 75 ms. A precursor target isolation window of 1.4 m/z was applied and a normalized collision energy of 35 was employed for fragmentation. Digested samples were resuspended in 30–60 μL of the mobile phase solvent A and then separated by reversed-phase chromatography using a Dionex Ultimate 3000 RSLC nanoUPLC system on a home-made 75 μm ID × 50 cm C18 capillary column (Reprosil-Pur AQ 120 Å, 1.9 μm). Peptides were separated by applying a non-linear 90 min gradient ranging from 99% of solvent A to 90% of solvent B (as above) at a flow rate of 250 nl/min. For quantitative analysis, digested samples were spiked with a known amount of heavy-labeled surrogate peptide standards (**Table 2**), ranging from 20–100 fmol/μL (final concentration). Typically, 4–8 μL of sample volumes were injected containing 160–800 fmol of heavy-labeled peptide standards. The amount of spiked heavy-labeled peptide corresponded to 20–50% of the relative abundance of the light endogenous peptides, as determined by preliminary PRM analyses. A single- *vs*. multiple spike-in heavy peptide standard concentration was used to calculate absolute levels of PceB protein. For all PRM analyses, samples were processed and analyzed using a single spike-in reference standard at the concentration mentioned above. A PRM validation experiment was performed for PceB using a synthetic fragment of PceB (ProteoGenix, Schiltigheim, France) that was quantified with the BCA assay and digested as described above (see Supplementary Material for details).

### 2.10. Data Processing and Database Searches

PEAKS Studio X+ Pro software (Bioinformatics Solutions Inc.) was used for data processing. The raw MS data files were imported into PEAKS Studio software using the following parameters for the database search. For protein identification, the UniProt/Swiss-Prot *Dehalobacter restrictus* database (Proteome ID UP000018934) combined with a decoy database was used. For peptide identification, the following settings were used: enzyme: Trypsin; missed cleavages: 2; precursor mass tolerance: 10 ppm; fragment mass tolerance: 0.2 Da; minimum charge: 2; maximum charge: 5; fixed modifications: Carbamidomethyl (C); variable modifications: Oxidation (M). False discovery rate (FDR) was calculated based on the target/decoy database and peptides as well as proteins with FDR threshold of 1% were chosen as true positive hits. Quantitative data analyses were performed using Skyline (version 21.1.0.278, MacCoss lab, University of Washington, USA), an open source software tool application for quantitative data processing and proteomic analysis. All integrated peaks were manually inspected to ensure correct peak detection and integration. Protein concentrations were calculated using peaks area ratios (light/heavy) derived from accurately quantified and spiked-in heavy-isotope labeled peptide standards. The concentration of each protein of interest was calculated from the average of the concentrations of its quantified peptides in the technical triplicates. The standard deviation was calculated using the STDEV.S method in Excel.

## 3. Results

The present study investigates the electron-accepting moiety of organohalide respiration in *D. restrictus* and *D. hafniense* strain TCE1 by defining the stoichiometric relationships of the *pceABCT* individual gene products at RNA and protein levels. The investigation of our model organisms allowed us to explore the use of the *pceABCT* gene cluster in two different bacterial genera and to question the participation of the encoded proteins in the composition of the electron transfer chain involved in PCE reductive dechlorination. The transcription and co-transcription of individual genes from the *pceABCT* gene clusters were elucidated *via* a combination of reverse transcription (RT)-PCR and quantitative RT-PCR. At protein level, the stoichiometric relationships between the *pceABCT* encoded proteins was addressed *via* PRM quantitative proteomics.

### 3.1. The *pceABCT* Genes Form an Operon

**Figure 1:**
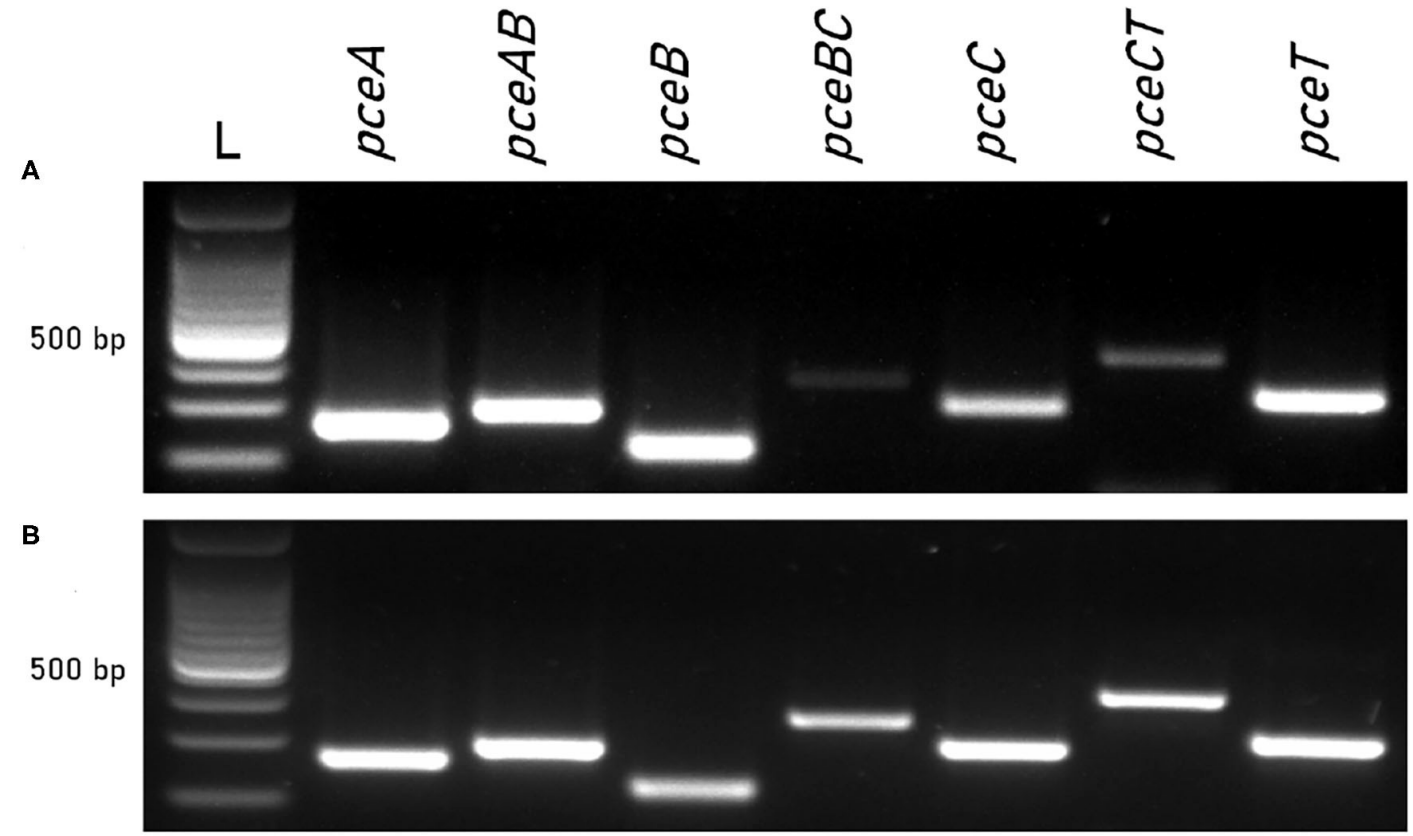
Co-transcription of *pce* genes in *D. restrictus* and *D. hafniense* strain TCE1. Individual genes and gene junctions were targeted by RT-PCR on RNA from **(A)** *D. restrictus* and **(B)** *D. hafniense* strain TCE1 cultivated with H_2_ and PCE as electron donor and acceptor, respectively.

The co-transcription of *pceABCT* individual genes was investigated in *D. restrictus* and *D. hafniense* strain TCE1 *via* RT-PCR. PCR primers targeting individual genes and also the 3′-end and 5′-end of successive gene pairs were applied to complementary DNA obtained from both strains cultivated in OHR conditions. It resulted in the transcription of the *pceABCT* individual genes as well as that of the different intergenic regions (Figure 1), revealing the operon nature of *pceABCT* gene clusters. A differential intensity of the PCR products was observed in *D. restrictus*, which was mostly evident in the lower abundance of the *pceBC* and *pceCT* intergenic regions (Figure 1A). This was not the case for *D. hafniense* strain TCE1 as all PCR products displayed a similar level of amplification (Figure 1B).

### 3.2. Stoichiometric Relationships of *pceABCT* Gene Products at RNA Level

**Figure 2:**
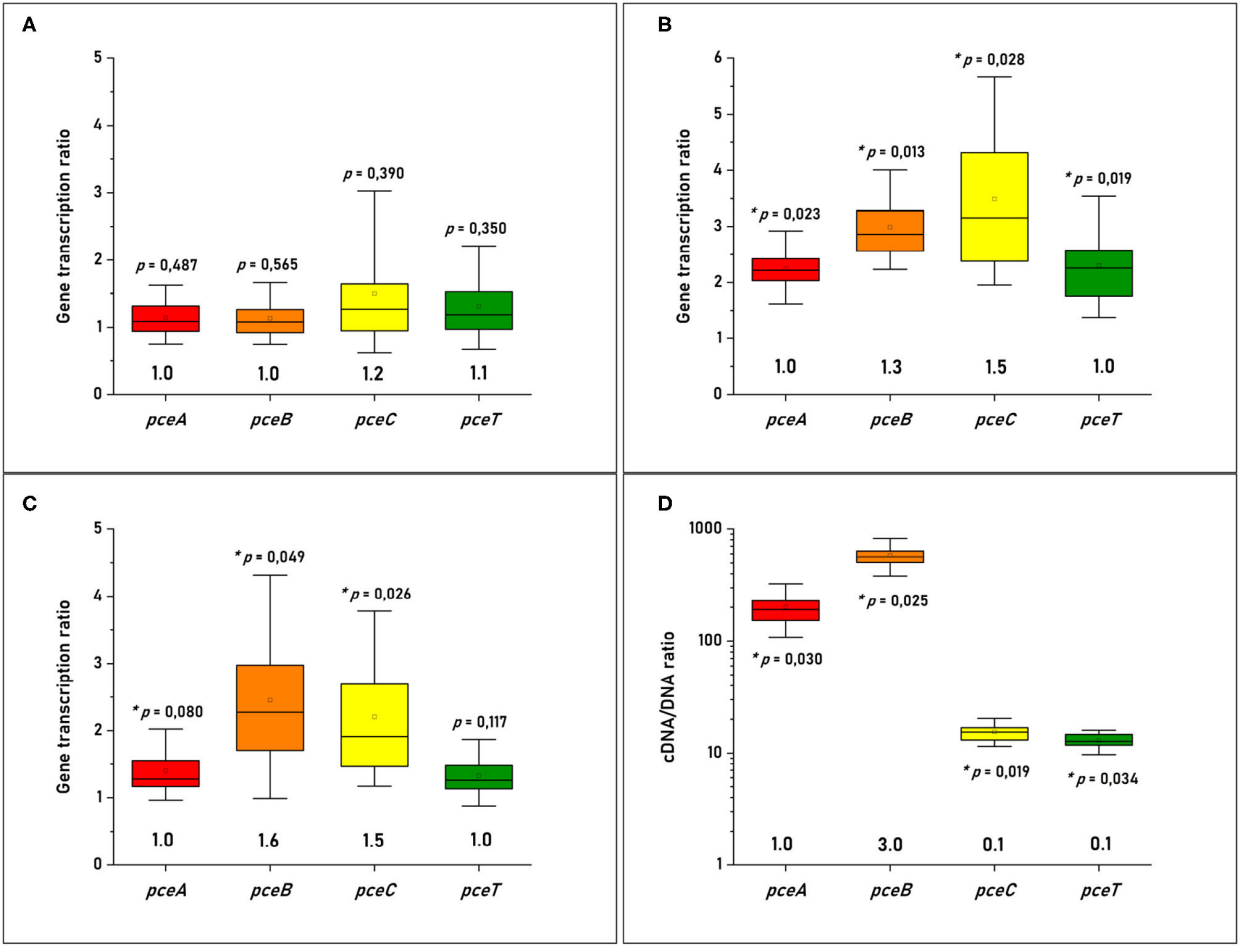
Quantitative analysis of the individual *pce* transcripts in **(A)** *D. hafniense* strain TCE1 cultivated on pyruvate and spiked with PCE; **(B)** *D. hafniense* strain TCE1 routinely cultivated with H_2_ and PCE; **(C)** *D. hafniense* strain TCE1 routinely cultivated with pyruvate and PCE; and **(D)** *D. restrictus* routinely cultivated with H_2_ and PCE. Please note that the transcriptional data of *D. hafniense* strain TCE1 were compared to transcripts obtained from cells routinely cultivated with pyruvate, while the gene transcription ratio of *D. restrictus* was calculated using DNA as reference.

The versatile energy metabolism of *D. hafniense* strain TCE1, in contrast to *D. restrictus*, allowed us to evaluate the transcription levels of the *pceABCT* genes in different growth conditions. To address the question of the stoichiometry of individual gene products at RNA level, fermentation with pyruvate and OHR metabolism were considered here. A spike of PCE to *D. hafniense* strain TCE1 cells growing fermentatively on pyruvate did not show any regulation of the *pce* genes when compared to non-spiked cells. The relative stoichiometry of the *pceABCT* genes was calculated to 1.0:1.0:1.2:1.1, after normalization of the data to the transcription level of *pceA* (Figure 2A). Then, the analysis was conducted on strain TCE1 cells routinely growing with hydrogen and PCE (H_2_-PCE), which revealed a slight, however significant, increase in the transcription level of all *pce* genes in comparison to fermentatively growing cells (Figure 2B) and revealed a normalized stoichiometry of 1.0:1.3:1.5:1.0. Similarly, a slight increase in the transcription level was also obtained mainly for *pceB* and *pceC* in cells routinely cultivated with pyruvate and PCE as electron donor and acceptor (Pyr-PCE), respectively, revealing a stoichiometry of 1.0:1.5:1.6:1.0 (Figure 2C). The RT-qPCR analysis conducted on *D. restrictus* cells was performed slightly differently as no spike nor alternative growth conditions could be applied (Figure 2B). There, the level of transcription of the *pce* genes was compared to the gene copy number in DNA obtained from the same samples during the RNA extraction procedure. The analysis of the transcription pattern of the *pce* genes revealed a clearly higher transcription level of *pceA* and *pceB* than that of *pceC* and *pceT* with a normalized relative stoichiometry of ~ 1.0:3.0:0.1:0.1 (Figure 2D).

### 3.3. Stoichiometry of Pce Proteins by Quantitative Proteomics

The analysis of *pceABCT* encoded proteins consisted of a combination of discovery proteome analysis (shotgun LC-MS/MS) and a quantitative proteomic analysis with heavy-labeled peptide standards. Both approaches were applied to the same samples, namely cell-free extracts and sub-cellular fractions of *D. restrictus* and *D. hafniense* strain TCE1. The discovery analysis was performed to evaluate the overall proteome and to define which specific peptides from the Pce proteins were best suited for the quantitative analysis with heavy-labeled reference peptides. The discovery analysis identified 1,433 proteins in total for *D. restrictus* across the different samples (corresponding to 50% of the genome-derived proteome), while 1751 proteins were detected in the proteome of *D. hafniense* strain TCE1 (35% of the genome-derived proteome). As showed in Table 1, this analysis allowed us the identification of the complete set of Pce proteins in both *D. restrictus* and *D. hafniense* strain TCE1, however with different outcome depending on the nature of the proteins and the type of samples. Furthermore, as example for *D. restrictus*, the analysis revealed a high level of detection of PceA which was characterized by a high sequence coverage (between 74 and 81%) and its position within the top 6 best identified proteins across the different samples when considering the total number of MS spectra (data not shown). A similar trend was observed for PceT, which displayed a sequence coverage in the range between 68 and 75% in the different fractions. However, overall PceT ranked slightly lower than PceA in the number of detected MS spectra. By contrast, the results on the two integral membrane proteins, namely PceB and PceC, were characterized by a generally lower detection level in the samples. For PceB, the highest sequence coverage (31%) was observed in the cell-free extract. Only two MS spectra identified PceB in the soluble fraction, possibly due to a slight cross-contamination during the fractionation of cell-free extracts. Finally, PceC was not detected in the soluble fraction, and showed only a limited sequence coverage (mainly from the FMN-binding domain, see Supplementary Figure 2) and low numbers of MS spectra in both the cell-free extract and the membrane fraction. Overall, a similar pattern was observed for the detection and distribution of the PceA, B, C, and T proteins in the discovery analysis of *D. hafniense* strain TCE1. Despite the known limitations in the detection of integral membrane proteins in general, and of PceB and PceC in particular, this dataset helped us to define several peptide candidates to be targeted by the quantitative analysis.

**Table 1:** Discovery MS analysis on cell-free extracts, soluble and membrane fractions of *D. restrictus* and of *D. hafniense* strain TCE1.

***D. restrictus*****/*****D. hafniense*** **strain TCE1^a^**				
**Protein**	**Sample**	**Coverage (%)**	**# MS spectra^b^**	**Area^c^**
PceA	Cell-free extract	81 / 69	436 / 294	2·10^10^ / 3·10^9^
	Soluble fraction	74 / 74	259 / 182	7·10^9^ / 6·10^9^
	Membrane fraction	78 / 85	418 / 487	2·10^10^ / 2·10^10^
PceB	Cell-free extract	41 / 34	28 / 40	6·10^8^ / 1·10^9^
	Soluble fraction	19 / -	2 / -	2·10^6^ / -
	Membrane fraction	32 / 41	34 / 27	4·10^8^ / 5·10^8^
PceC	Cell-free extract	24 / 16	15 / 9	1·10^8^ / 3·10^7^
	Soluble fraction	- / 2	- / 1	- / 2·10^6^
	Membrane fraction	22 / 25	14 / 22	1·10^8^ / 2·10^8^
PceT	Cell-free extract	72 / 68	100 / 72	6·10^9^ / 4·10^8^
	Soluble fraction	75 / 90	122 / 233	6·10^9^ / 1·10^10^
	Membrane fraction	62 / 82	56 / 60	2·10^9^ / 8·10^8^
ATP synthase	Cell-free extract	74 / 29	119 / 23	7·10^9^ / 1·10^8^
	Soluble fraction	68 / 39	83 / 29	2·10^9^ / 6·10^8^
	Membrane fraction	76 / 57	146 / 50	6·10^9^ / 8·10^8^

a *For each data entry, the first value corresponds to the proteomic analysis of D. restrictus and the second to that of D. hafniense strain TCE1*.

b *# MS spectra correspond to the total number of spectra identified that support the given protein*.

c *Area is the total area or intensity of peptide features from unique supporting peptides of the corresponding protein. This can be used as an indicator of the abundance of the protein*.

The investigation of stoichiometry was conducted on cell-free extracts from biomass harvested at exponential phase. In addition, to define the relative abundance of the targeted proteins in the sub-cellular compartments, the quantification was also performed on the soluble and membrane fractions issued from cell-free extracts following a protocol that minimized protein extraction biases. RapiGest-based extraction was applied to whole membrane fractions without separating the proteins from the membrane particles. Although we cannot exclude that some of the proteins would remain embedded in the membrane lipids upon extraction, we hypothesize that the solvent-exposed fragments of these proteins are likely accessible to and digested by trypsin, thus minimizing their loss during PRM analysis. At this stage, whether the protocol applied here failed to detect part of the membrane proteins cannot be evaluated, and thus represents a certain limitation in our analysis. The selection of signature peptides based on the criteria reported in the Material and Methods section resulted in the definition of a total of 16 unique peptides (Table 2). Based on first results, a shorter selection of 10 labeled peptides was established (indicated in bold in Table 2), corresponding to a minimum of two peptides for each protein of interest, with the exception of PceB that could only be targeted by one peptide due to the small size and hydrophobic nature of the protein and due to discrepancies in the detection of the second peptide in the preliminary analyses. An example of the obtained PRM spectra is given in Supplementary Figure 3. For PceB, due to the overall scarcity and low solubility of its peptides, we sought to validate the reproducible quantification of the single PceB tryptic peptide LANHPAK by assessing the digestibility and recovery of this peptide, using a synthetic, quantified form of the C-terminal part of PceB, spanning the sequence of amino acid residues from 63 to 105. We show that this peptide is indeed a reproducible cleavage product of the PceB protein and therefore serves a surrogate reference peptide for the quantification of PceB in biological samples. PRM applied to the synthetic PceB protein fragment with spike-in heavy-labeled peptide revealed a 19% difference in PceB concentration. This value seems acceptable considering the experimental variation of the PRM analysis, as well as that of the initial quantification of the synthetic PceB protein. In addition, LC-MS/MS analysis on the synthetic PceB fragment confirmed that the selected peptide was the most abundant cleavage product found, with other mis-cleaved forms (-KK) or truncated forms of PceB representing less than 1% (relative peak area) of the main proteolytic product (Supplementary Figure 4). Moreover, this peptide has been predicted to protrude out of the cytoplasmic membrane (Schubert et al., 2018), and is likely to be relatively accessible for trypsin digestion.

**Table 2:** List of heavy-labeled peptides.

**Peptide #**	**Species**	**Sequence**	**Mass [m/z] Light/Heavy**
1^*^	PceA	**YLPWDLPK**	516.28166 / 520.28876
2^**^	PceA	**TSPSVISSATVGK**	617.33789 / 621.34499
3	PceA	IATQIPLLQDAAR	705.40918 / 710.41332
4	PceA	**LLGADLVGIAPYDER**	801.43031 / 806.43445
5	PceB	GGTALIPAIITYR	673.39554 / 678.39968
6	PceB	**LANHPAK**	375.71649 / 379.72359
7	PceC	**NVLGVISIEK**	536.32406 / 540.33116
8	PceC	**QGETPVFFER**	605.29857 / 610.3027
9	PceC	EPIYLGGAYGYSGYLGSIK	1004.50675 / 1008.51385
10	PceC	YFDGFQGLAIK	629.82696 / 633.83406
11	PceT	**EVSANLLGK**	465.76637 / 469.77347
12	PceT	WWGSEFTFTVK	694.3377 / 698.34479
13	PceT	**DATVPVIR**	435.75581 / 440.75994
14	ATP synthase	**ELIIGDR**	408.2377 / 413.2388
15	ATP synthase	ELSLLLK	408.2655 / 412.2726
16	ATP synthase	**QVAGQLR**	386.2272 / 391.2314

For each peptide, the labeled amino acid is marked in red. All the indicated mass-to-charge (m/z) ratios are given for a peptide charge state of 2. Reference peptides used for PRM analysis are indicated in bold. Indicated with a star (^*^) is the peptide used for D. hafniense strain TCE1, while (^**^) indicates is the alternative peptide used for D. restrictus.

**Figure 3:**
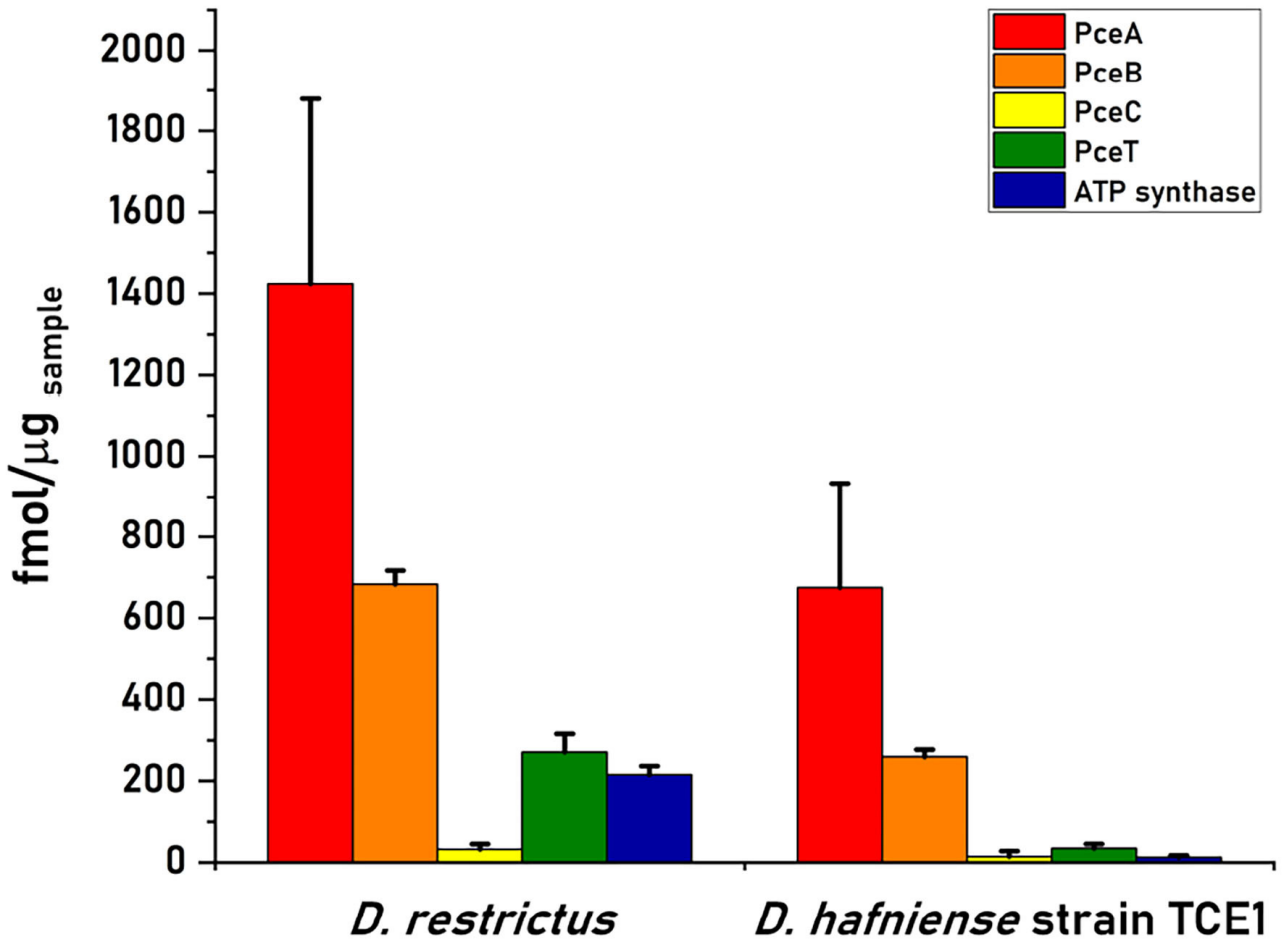
Quantitative proteomics analysis of Pce proteins and the F1 α-subunit of the ATP synthase in cell-free extracts (CFE) from *D. restrictus* and *D. hafniense* strain TCE1. This graph shows the results of one biological replicate and is representative of the trend also observed for the second replicate (see Supplementary Table 2). The concentration of each protein was calculated by averaging the values obtained for the selected peptides in technical triplicates. Error bars indicate the calculated standard deviation.

**Figure 4:**
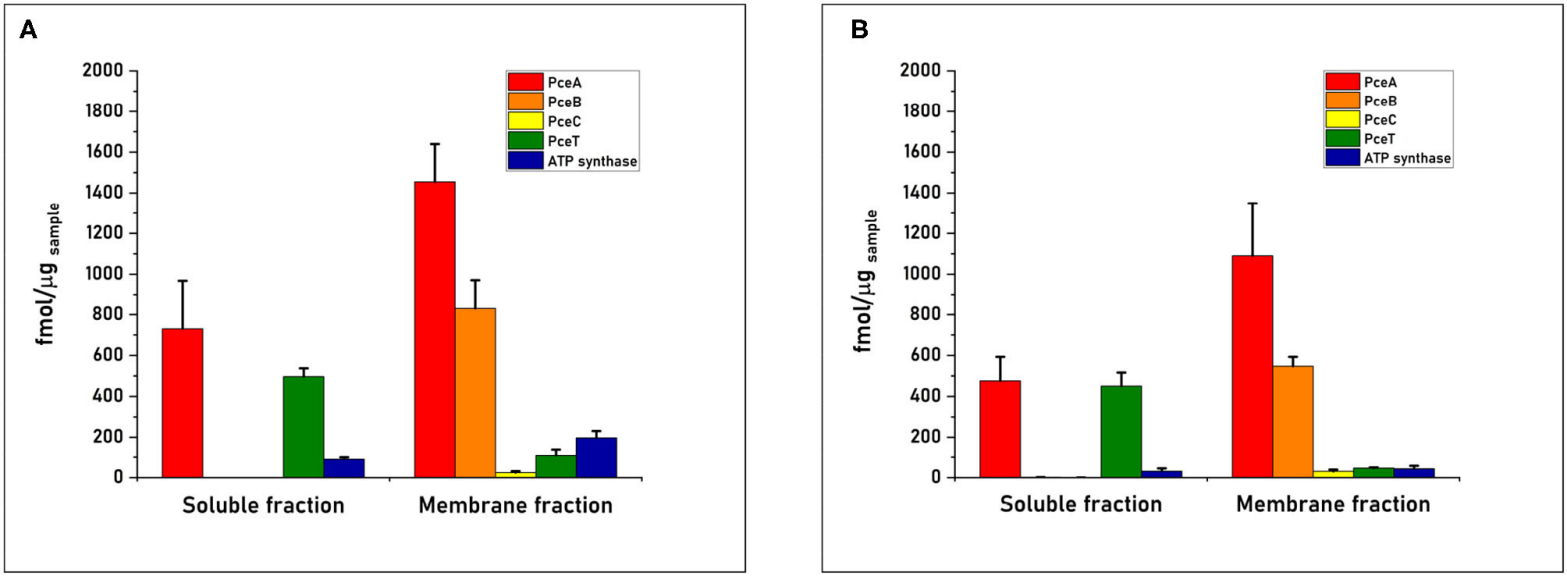
Quantitative proteomics of the Pce proteins and the F1 α-subunit of the ATP synthase in sub-cellular compartments obtained from cell-free extracts of **(A)** *D. restrictus* and **(B)** *D. hafniense* strain TCE1. These graphs shows the results of one biological replicate and are representative of the trend also observed for the second replicate (see Supplementary Table 2). The concentration of each protein was calculated by averaging the values obtained for the selected peptides in technical triplicates. Error bars indicate the calculated standard deviation.

The quantification of PceA, B, C, and T proteins from the cell-free extract of *D. restrictus* resulted in a relative stoichiometry normalized to PceA of 1.0:0.5:0.02:0.2, showing a likely 2:1 stoichiometry between PceA and PceB, while PceC and PceT resulted ~ in 50- and 5-fold lower abundance than PceA, respectively (Figure 3). In *D. hafniense* strain TCE1, the cell-free extract displayed a slightly different ratio (1:0.38:0.02:0.05) with a calculated PceA/PceB ratio of ~ 3:1. PceC and PceT resulted in a 50- and 20-fold lower abundance than PceA, respectively, similarly to *D. restrictus* (Figure 3). In the soluble fraction of *D. restrictus*, PceA and PceT were largely present while PceB and PceC could not be detected. The data thus revealed a 1.0:0.8 stoichiometry between PceA and PceT (Figure 4A). A similar trend was also observed in the soluble fraction of *D. hafniense* strain TCE1, displaying a PceA:PceT stoichiometry of 1.0:1.0 (Figure 4B). In the membrane fraction, the analysis showed a stoichiometry of 1.0:0.57:0.02:0.07 across the Pce proteins in *D. restrictus* (Figure 4A), while *D. hafniense* strain TCE1 displayed a ratio of 1.0:0.5:0.03:0.04 (Figure 4B). Overall, the results revealed a similar trend in the membrane fraction of both strains, with PceA and PceB resulting as predominant subunits and exhibiting a relative stoichiometry of 2:1 in favor of PceA. As observed in cell-free extracts, both PceC and PceT resulted as many times less abundant proteins than PceA. The results shown in Figures 3, 4 are derived from data of one biological replicate for each strain and is representative of the trend also observed for the second biological replicate (Supplementary Table 2). The nature of integral membrane proteins and their challenging quantification invite us to consider these results with care and to propose a physiological and biochemical interpretation which may require additional evidence in the future.

## 4. Discussion

Reductive dehalogenase (*rdh*) genes are typically organized in clusters composed of at least *rdhA*, the gene encoding the key catalytic enzyme, and *rdhB*, a gene encoding the putative membrane anchor protein for RdhA. This minimal gene set, however, is frequently accompanied by a variable set of accessory genes, for most of which the exact function is still unknown (Maillard and Willemin, 2019). In the present study, the global expression of the *pce* genes from the conserved *pceABCT* gene clusters was investigated *via* a multilevel approach on RNA and proteins.

### 4.1. Transcription Pattern Heterogeneity and Stoichiometry of Gene Transcripts Associated With *rdh* Clusters in the Firmicutes

During the last two decades many studies have addressed the transcription of *rdh* genes in OHRB by applying a large variety of methodologies (Türkowsky et al., 2018). Among them, only a few studies have reported on the transcription of *rdhA*-associated genes. The co-transcription of *rdhAB* or *rdhBA* gene pairs has been reported for *S. multivorans* (Neumann et al., 1998), *Dehalococcoides* spp. (Mukherjee et al., 2014; Padilla-Crespo et al., 2014) and *D. dichloroeliminans* (Marzorati et al., 2007), while only three studies have been conducted on *rdh* accessory genes, namely the *cprTKZEBACD* gene cluster of *D. dehalogenans*, the *pceABCT* gene cluster of *D. hafniense* strain Y51, and the OHR gene region in *S. multivorans* (Smidt et al., 2000; Futagami et al., 2006; Esken et al., 2020). In *D. dehalogenans* a combination of Northern blot and RT-PCR has revealed a strong regulation of the *cpr* gene cluster in presence of the organohalogen 3-chloro-4-hydroxyphenylacetate, resulting in the transcription of bicistronic units, i.e., *cprZE, cprBA*, and *cprCD*, along with occasional formation of the polycistronic *cprBACD* transcript (Smidt et al., 2000). In the work on the *pce* cluster harbored by *D. hafniense* strain Y51, Northern blot analysis has shown the co-transcription of *pceABC*, while *pceT* was transcribed as a monocistronic transcript. RT-PCR analysis, on the other hand, has suggested a co-transcription of *pceC* and *pceT*, thus challenging the Northern blot results (Futagami et al., 2006). Finally, the transcriptomic analysis of the OHR region in *S. multivorans* has confirmed the co-transcription of *pceA* and *pceB* genes and also revealed the PCE-dependent up-regulation of several transcriptional units among which a 29-gene transcript including the putative quinol dehydrogenase *pceMN* genes and the corrinoid biosynthesis genes (Esken et al., 2020). In the present work, the transcription pattern of *rdh* genes observed in *D. restrictus* showed the co-transcription of all four of the *pce* gene targets, which led us to consider *pceABCT* as an operon. However, the stoichiometry analysis of *pce* individual gene transcripts in *D. restrictus* displayed a significantly higher level of transcription of *pceA* and *pceB*, as it appeared ten- and thirty-fold more abundant than that of *pceC* and *pceT*, respectively. A different transcription pattern was observed for *D. hafniense* strain TCE1 cells growing fermentatively on pyruvate and subsequently spiked with PCE. Indeed, the quantitative analysis displayed a comparable abundance of all *pce* gene transcripts after the spike. Collectively, these results confirm the lack of transcriptional regulation of the *pce* gene cluster in *D. hafniense* strain TCE1, which is likely due to the presence of a constitutive strong promoter upstream of *pceA* that is encoded in the right inverted repeat of the *IS*Dha1 insertion sequence, as already proposed in previous studies (Maillard et al., 2005; Duret et al., 2012). In addition, the higher level of transcription of *pceAB* observed in *D. restrictus*, but not in *D. hafniense* strain TCE1, raises new questions on possible post-transcriptional events occurring in the operon, such as RNA processing and differential RNA stability (Trinquier et al., 2020). This is possibly corroborated by sequence analysis of the *pceABCT* operons from *D. restrictus* and *D. hafniense* strain TCE1, revealing a predicted hairpin loop structure in the *pceBC* intergenic region (Supplementary Figure 1B). One possible scenario is that the hairpin loop may protect the 3′-end of the *pceAB* transcript from degradation by 3'-exoribonucleases once the *pceABCT* mRNA is processed into two or more fragments, while the *pceCT* transcript is partially degraded by exoribonucleases. The data obtained for *D. hafniense* strain TCE1 contrast with this hypothesis, but one explanation for this difference might be found in specific pools of RNA processing enzymes in *D. restrictus* and *D. hafniense*. The abundance of *pce* gene transcripts, however, cannot be used as proxy for the abundance of the cognate proteins (for a review see Liu et al., 2016).

### 4.2. Challenges in the Detection of Rdh Proteins From Firmicutes

Focusing exclusively on OHRB belonging to the Firmicutes, proteomic analyses of Rdh proteins were reported in twelve studies (Prat et al., 2011; Rupakula et al., 2013, 2015; Tang and Edwards, 2013; Kruse et al., 2015; Jugder et al., 2016; Alfán-Guzmán et al., 2017; Kleindienst et al., 2019; Liu et al., 2019; Low et al., 2019; Peng et al., 2020; Chen et al., 2021). The different methodologies that were applied in those studies (1 or 2D native or denaturing gels, and in-solution LC-MS/MS analyses) render their comparison very difficult. Nevertheless, one common feature is the challenge to detect integral membrane proteins as their extraction often results in low abundance. Indeed, they are (at least partially) embedded in membrane lipids, and their hydrophobic transmembrane helices are known to be relatively poor in the trypsin-targeted arginine and lysine residues. From these studies, it is clear that the integral membrane proteins RdhB and RdhC were less frequently detected than the membrane-associated RdhA proteins (Supplementary Table 3). On the one hand, the small and highly hydrophobic RdhB was only detected in one third of these studies, while RdhC, that displays an exposed peripheral domain (Buttet et al., 2018), was identified in half of the relevant studies (4 out of 8). The cytoplasmic RdhT molecular chaperone was identified in two-thirds of the relevant studies (4 out of 6), the two missing cases can be explained by the methodology applied (selected in-gel analysis). The results of our discovery proteomic analysis seem to be in line with these considerations since PceA and PceT were detected with a higher coverage, as well as a higher number of MS spectra and their derived total area, than PceB and PceC (Table 1). Whether the issue regarding the detectability of integral membrane proteins precludes, or at least, bias the comparison of proteins of very different nature needs to be carefully addressed. This issue was also the reason why we decided to apply a quantitative approach with selected heavy-labeled reference peptides.

### 4.3. PceA and PceB - but Not PceC - Appear With a Similar Concentration in the Membrane Fraction

Based on physiological data and sequence information reported for the *pceABCT* operon, a tentative model of the electron transfer chain involved in OHR has been proposed previously, where PceA, B, and C could form a membrane-bound protein complex (Maillard and Holliger, 2016). There, PceC was suggested to play a role in electron transfer in agreement with the redox activity of the FMN-binding domain (Buttet et al., 2018). In this context, the elucidation of the stoichiometry of the Pce proteins present in membrane fractions constitutes an important information. The application of PRM-based quantitative proteomics succeeded to detect all Pce proteins and to quantify them in *D. restrictus* and *D. hafniense* strain TCE1, thus offering a new and presumably more accurate vision of the biochemical premise for the terminal reductase involved in (at least some) OHRB. The analysis of cell-free extracts revealed an apparent stoichiometry of PceA to PceB of 2:1 or 3:1, while PceC and PceT were less abundant. It was especially pronounced for PceC, which is in agreement with recent studies on the *rdhABC* gene cluster of *Desulfoluna* sp., where the C subunit was not detected at all (Liu et al., 2019; Peng et al., 2020). In the soluble fraction, a 1:1 ratio between PceA and PceT suggests that most of the PceA that is not yet associated with the membrane is likely accompanied by PceT, giving additional evidence of the role of PceT as a molecular chaperone specifically dedicated to the maturation of PceA (Morita et al., 2009; Maillard et al., 2011). At the membrane, an apparent stoichiometry of 2:1 between PceA and PceB was identified in both strains, while again PceC and PceT were largely under-represented. In spite of the challenges that integral membrane proteins pose to their quantification, we think that the measured stoichiometry of the Pce proteins better reflects the physiological state than any previous analysis based on non-quantitative proteomics. The PceA:PceB ratio in the membrane fraction is clearly improved in favor of PceB when compared to the ratio estimated from our discovery MS analysis (Table 1) and to that of a previous study (Rupakula et al., 2013). The possibility that, due to its hydrophobic nature, PceB remains largely undetected in our quantitative analysis has no clear support. Although we cannot unambiguously exclude this possibility, the observed PceA:PceB stoichiometry is rather in agreement with the well-accepted function of PceB as membrane anchor for PceA and the proposed 1:1 interaction of both proteins in the membrane (Bommer et al., 2014; Wang et al., 2018). Moreover, the targeted PceB peptide is predicted to protrude outside the cytoplasmic membrane and, thus, should be exposed to trypsin digestion. Taken together, and beyond possible limitations in the detection of integral membrane proteins, the stoichiometry results strengthen the mutual link between RdhA and RdhB proteins, thus corroborating the role of the B subunit in anchoring the catalytic subunit at the membrane, as already proposed in previous studies (Neumann et al., 1998; Smidt et al., 2000). The elucidation of the crystal structure of PceA from *S. multivorans* has identified PceA as a homodimer, which led to the proposition of a Pce(AB)_2_ complex associated with the cytoplasmic membrane (Bommer et al., 2014). In agreement with the dimeric structure of PceA, the observed 2:1 stoichiometry between PceA and PceB identified here rather suggests that the dimeric PceA is attached to the membrane by only one copy of PceB in a possible PceA_2_B protein complex.

## 5. Conclusions

Overall, the present study showed a strong relationship between *pceA* and *pceB* gene products, both at RNA and protein levels, thus demonstrating the mutual importance of these two subunits for the OHR metabolism. For the first time, a quantitative proteomics approach targeting the key proteins in OHR helped us to challenge the model for the electron-accepting moiety in some Firmicutes (Maillard and Holliger, 2016) and to propose a new putative PceA_2_B reductive dehalogenase complex associated with the cytoplasmic membrane, thus likely excluding PceC from the complex. It is conceivable that PceC plays the role of a helper protein toward PceA, thus not fully excluding its transient and sub-stoichiometric participation in the reductive dehalogenase complex. Further biochemical investigation is needed to unambiguously confirm our results and to elucidate the exact composition of the electron-transfer chain involved in PCE dechlorination in *D. restrictus* and *D. hafniense* strain TCE1.

## Data Availability Statement

The datasets presented in this study can be found in online repositories. The names of the repository/repositories and accession number(s) can be found in the article.

## Author Contributions

JM and LC conceived the study and designed the experiments. LC performed the experiments, analyzed the data, and wrote the manuscript. AS performed mass spectrometry analysis. JM performed experiments and corrected the manuscript. CH advised in the experimental strategy and the analysis of the data and corrected the manuscript. All authors contributed to the article and approved the submitted version.

## Funding

The research was funded by the Swiss National Science Foundation (SNSF) in the frame of the SNF Project No. 31003A_173059.

## Conflict of Interest

The authors declare that the research was conducted in the absence of any commercial or financial relationships that could be construed as a potential conflict of interest.

## Publisher's Note

All claims expressed in this article are solely those of the authors and do not necessarily represent those of their affiliated organizations, or those of the publisher, the editors and the reviewers. Any product that may be evaluated in this article, or claim that may be made by its manufacturer, is not guaranteed or endorsed by the publisher.

## References

[B1] Alfán-Guzmán R. Ertan H. Manefield M. Lee M. (2017). Isolation and characterization of *Dehalobacter* sp. strain TeCB1 including identification of TcbA: A novel tetra- and trichlorobenzene reductive dehalogenase. Front. Microbiol. 8, 558. 10.3389/fmicb.2017.00558 28421054 PMC5379058

[B2] Bommer M. Kunze C. Fesseler J. Schubert T. Diekert G. Dobbek H. (2014). Structural basis for organohalide respiration. Science 346, 455–458. 10.1126/science.1258118 25278505

[B3] Buttet G. F. Willemin M. S. Hamelin R. Rupakula A. Maillard J. (2018). The membrane-bound C subunit of reductive dehalogenases: topology analysis and reconstitution of the FMN-binding domain of PceC. Front. Microbiol. 9, 755. 10.3389/fmicb.2018.00755 29740408 PMC5928378

[B4] Chen G. Jiang N. Solis M. I. V. Murdoch F. K. Murdoch R. W. Xie Y.. (2021). Anaerobic microbial metabolism of dichloroacetate. mBio. 12, e00537-21. 10.1128/mBio.00537-21 33906923 PMC8092247

[B5] Comensoli L. Maillard J. Albini M. Sandoz F. Junier P. Joseph E. (2017). Use of bacteria to stabilize archaeological iron. Appl. Environ. Microbiol. 83, e03478–16. 10.1128/AEM.03478-16 28283522 PMC5394308

[B6] Duret A. Holliger C. Maillard J. (2012). The physiological opportunism of *Desulfitobacterium hafniense* strain TCE1 towards organohalide respiration with tetrachloroethene. Appl. Environ. Microbiol. 78, 6121–6127. 10.1128/AEM.01221-12 22729540 PMC3416639

[B7] Esken J. Goris T. Gadkari J. Bischler T. Förstner K. U. Sharma C. M.. (2020). Tetrachloroethene respiration in *Sulfurospirillum* species is regulated by a two-component system as unraveled by comparative genomics, transcriptomics, and regulator binding studies. MicrobiologyOpen 9, e1138. 10.1002/mbo3.1138 33242236 PMC7755780

[B8] Futagami T. and Furukawa, K.. (2016). The genus *Desulfitobacterium*, in Organohalide-Respiring Bacteria, eds L. Adrian, and F. E. Löffler (Heidelberg: Springer), 173–207.

[B9] Futagami T. Yamaguchi T. Nakayama S.-I. Goto M. Furukawa K. (2006). Effects of chloromethanes on growth of and deletion of the pce gene cluster in dehalorespiring *Desulfitobacterium hafniense* strain Y51. Appl. Environ. Microbiol. 72, 5998–6003. 10.1128/AEM.00979-06 16957221 PMC1563609

[B10] Gerritse J. Drzyzga O. Kloetstra G. Keijmel M. Wiersum L. P. Hutson R.. (1999). Influence of different electron donors and acceptors on dehalorespiration of tetrachloroethene by *Desulfitobacterium frappieri* TCE1. Appl. Environ. Microbiol. 65, 5212–5221. 10.1128/AEM.65.12.5212-5221.1999 10583967 PMC91707

[B11] Goris T. Schiffmann C. L. Gadkari J. Schubert T. Seifert J. Jehmlich N.. (2015). Proteomics of the organohalide-respiring epsilonproteobacterium *Sulfurospirillum multivoran*s adapted to tetrachloroethene and other energy substrates. Sci. Rep. 5, 13794. 10.1038/srep13794 26387727 PMC4585668

[B12] Holliger C. Hahn D. Harmsen H. Ludwig W. Schumacher W. Tindall B.. (1998). *Dehalobacter restrictus* gen. nov. and sp. nov., a strictly anaerobic bacterium that reductively dechlorinates tetra- and trichloroethene in an anaerobic respiration. Arch. Microbiol. 169, 313–321. 10.1007/s002030050577 9531632

[B13] Hug L. A. Maphosa F. Leys D. Löffler F. E. Smidt H. Edwards E. A.. (2013). Overview of organohalide-respiring bacteria and a proposal for a classification system for reductive dehalogenases. Philosoph. Trans. R. Soc. B Biol. Sci. 368, 20120322. 10.1098/rstb.2012.0322 23479752 PMC3638463

[B14] Jugder B.-E. Ertan H. Wong Y. K. Braidy N. Manefield M. Marquis C. P.. (2016). Genomic, transcriptomic and proteomic analyses of *Dehalobacter* UNSWDHB in response to chloroform. Environ. Microbiol. Rep. 8, 814–824. 10.1111/1758-2229.12444 27452500

[B15] Kleindienst S. Chourey K. Chen G. Murdoch R. W. Higgins S. A. Iyer R.. (2019). Proteogenomics reveals novel reductive dehalogenases and methyltransferases expressed during anaerobic dichloromethane metabolism. Appl. Environ. Microbiol. 85, e02768-18. 10.1128/AEM.02768-18 30658979 PMC6414379

[B16] Kruse T. Goris T. Maillard J. Woyke T. Lechner U. de Vos W.. (2017). Comparative genomics of the genus *Desulfitobacterium*. FEMS Microbiol. Ecol. 93, fix135. 10.1093/femsec/fix135 29040502

[B17] Kruse T. Maillard J. Goodwin L. Woyke T. Teshima H. Bruce D.. (2013). Complete genome sequence of *Dehalobacter restrictus* PER-K23 T. Stand. Gen. Sci. 8, 375–388. 10.4056/sigs.3787426 24501624 PMC3910700

[B18] Kruse T. van de Pas B. A. Atteia A. Krab K. Hagen W. R. Goodwin L.. (2015). Genomic, proteomic, and biochemical analysis of the organohalide respiratory pathway in *Desulfitobacterium dehalogenans*. J. Bacteriol. 197, 893–904. 10.1128/JB.02370-14 25512312 PMC4325113

[B19] Liu J. Adrian L. Häggblom M. M. (2019). Transcriptomic and proteomic responses of the organohalide-respiring bacterium *Desulfoluna spongiiphila* to growth with 2,6-dibromophenol as the electron acceptor. Appl. Environ. Microbiol. 86, e02146-19. 10.1128/AEM.02146-19 31836581 PMC7028966

[B20] Liu Y. Beyer A. Aebersold R. (2016). On the dependency of cellular protein levels on mRNA abundance. Cell 165, 535–550. 10.1016/j.cell.2016.03.014 27104977

[B21] Low A. Zhao S. Rogers M. J. Zemb O. Lee M. He J.. (2019). Isolation, characterization and bioaugmentation of an acidotolerant 1,2-dichloroethane respiring *Desulfitobacterium* species from a low pH aquifer. FEMS Microbiol. Ecol. 95, fiz055. 10.1093/femsec/fiz055 30980656

[B22] Maillard J. Genevaux P. Holliger C. (2011). Redundancy and specificity of multiple trigger factor chaperones in *Desulfitobacteria*. Microbiology 157, 2410–2421. 10.1099/mic.0.050880-0 21622524

[B23] Maillard J. Holliger C. (2016). The genus *Dehalobacter*, in Organohalide-Respiring Bacteria, eds L. Adrian and F. E. Löffler (Heidelberg: Springer), 153–171.

[B24] Maillard J. Regeard C. Holliger C. (2005). Isolation and characterization of *Tn*-dha1, a transposon containing the tetrachloroethene reductive dehalogenase of *Desulfitobacterium hafniense* strain TCE1. Environ. Microbiol. 7, 107–117. 10.1111/j.1462-2920.2004.00671.x 15643941

[B25] Maillard J. Schumacher W. Vazquez F. Regeard C. Hagen W. R. Holliger C. (2003). Characterization of the corrinoid iron-sulfur protein tetrachloroethene reductive dehalogenase of *Dehalobacter restrictus*. Appl. Environ. Microbiol. 69, 628–4638. 10.1128/AEM.69.8.4628-4638.2003 12902251 PMC169082

[B26] Maillard J. Willemin M. S. (2019). Regulation of organohalide respiration, in Advances in Microbial Physiology, Vol. 74, 191–238. 31126531 10.1016/bs.ampbs.2019.02.002

[B27] Marzorati M. Ferra F. D. Raemdonck H. V. Borin S. Allifranchini E. Carpani G.. (2007). A novel reductive dehalogenase, identified in a contaminated groundwater enrichment culture and in *Desulfitobacterium dichloroeliminans* strain DCA1, is linked to dehalogenation of 1,2-dichloroethane. Appl. Environ. Microbiol. 73, 2990–2999. 10.1128/AEM.02748-06 17351102 PMC1892866

[B28] Morita Y. Futagami T. Goto M. Furukawa K. (2009). Functional characterization of the trigger factor protein PceT of tetrachloroethene-dechlorinating *Desulfitobacterium hafniense* Y51. Appl. Microbiol. Biotechnol. 83, 775–781. 10.1007/s00253-009-1958-z 19347335

[B29] Mukherjee K. Bowman K. S. Rainey F. A. Siddaramappa S. Challacombe J. F. Moe W. M. (2014). *Dehalogenimonas lykanthroporepellens* BL-DC-9T simultaneously transcribes many *rdhA* genes during organohalide respiration with 1,2-DCA, 1,2-DCP, and 1,2,3-TCP as electron acceptors. FEMS Microbiol. Lett. 354, 111–118. 10.1111/1574-6968.12434 24673292

[B30] Neumann A. Wohlfarth G. Diekert G. (1998). Tetrachloroethene dehalogenase from *Dehalospirillum multivorans*: cloning, sequencing of the encoding genes, and expression of the *pceA* gene in *Escherichia coli*. J. Bacteriol. 180, 4140–4145. 9696761 10.1128/jb.180.16.4140-4145.1998 PMC107409

[B31] Ni S. Fredrickson J. K. Xun L. (1995). Purification and characterization of a novel 3-chlorobenzoate-reductive dehalogenase from the cytoplasmic membrane of *Desulfomonile tiedjei* DCB-1. J. Bacteriol. 177, 5135–5139. 7665493 10.1128/jb.177.17.5135-5139.1995 PMC177294

[B32] Oren A. Garrity G. M. . (2021). Valid publication of the names of forty-two phyla of prokaryotes. Int. J. Syst. Evol. Microbiol. 71, 005056. 10.1099/ijsem.0.005056 34694987

[B33] Padilla-Crespo E. Yan J. Swift C. Wagner D. D. Chourey K. Hettich R. L.. (2014). Identification and environmental distribution of *dcpA*, which encodes the reductive dehalogenase catalyzing the dichloroelimination of 1,2-dichloropropane to propene in organohalide-respiring chloroflexi. Appl. Environ. Microbiol. 80, 808–818. 10.1128/AEM.02927-13 24242248 PMC3911209

[B34] Peng P. Goris T. Lu Y. Nijsse B. Burrichter A. Schleheck D.. (2020). Organohalide-respiring *Desulfoluna* species isolated from marine environments. ISME J. 14, 815–827. 10.1038/s41396-019-0573-y 31896791 PMC7031245

[B35] Perez-Riverol Y. Csordas A. Bai J. Bernal-Llinares M. Hewapathirana S. Kundu D. J.. (2019). The PRIDE database and related tools and resources in 2019: improving support for quantification data. Nucl. Acids Res. 47, D442–D450. 10.1093/nar/gky1106 30395289 PMC6323896

[B36] Prat L. Maillard J. Grimaud R. Holliger C. (2011). Physiological adaptation of *Desulfitobacterium hafniense* strain TCE1 to tetrachloroethene respiration. Appl. Environ. Microbiol. 77, 3853–3859. 10.1128/AEM.02471-10 21478312 PMC3127588

[B37] Reinhold A. Westermann M. Seifert J. (2012). Impact of vitamin B12 on formation of the tetrachloroethene reductive dehalogenase in *Desulfitobacterium hafniense* strain Y51. Appl. Environ. Microbiol. 78, 8025–8032. 10.1128/AEM.02173-12 22961902 PMC3485949

[B38] Rupakula A. Kruse T. Boeren S. Holliger C. Smidt H. Maillard J. (2013). The restricted metabolism of the obligate organohalide respiring bacterium *Dehalobacter restrictus*: lessons from tiered functional genomics. Philosoph. Trans. R. Soc. B Biol. Sci. 368, 20120325. 10.1098/rstb.2012.0325 23479754 PMC3638465

[B39] Rupakula A. Lu Y. Kruse T. Boeren S. Holliger C. Smidt H.. (2015). Functional genomics of corrinoid starvation in the organohalide-respiring bacterium *Dehalobacter restrictus* strain PER-K23. Front. Microbiol. 5,751. 10.3389/fmicb.2014.00751 25610435 PMC4285132

[B40] Schubert T. Adrian L. Sawers R. G. Diekert G. (2018). Organohalide respiratory chains: composition, topology and key enzymes. FEMS Microbiol. Ecol. 94, fiy035. 10.1093/femsec/fiy035 29718172

[B41] Schubert T. von Reuß S. H. Kunze C. Paetz C. Kruse S. Brand-Schön P.. (2019). Guided cobamide biosynthesis for heterologous production of reductive dehalogenases. Microb. Biotechnol. 12, 346–359. 10.1111/1751-7915.13339 30549216 PMC6389850

[B42] Schumacher W. Holliger C. (1996). The proton/electron ratio of the menaquinone-dependent electron transport from dihydrogen to tetrachloroethene in *Dehalobacter restrictus*. J. Bacteriol. 178, 2328–2333. 10.1128/jb.178.8.2328-2333.1996 8636034 PMC177941

[B43] Seidel K. Kühnert J. Adrian L. (2018). The complexome of *Dehalococcoides mccartyi* reveals its organohalide respiration-complex is modular. Front. Microbiol. 9,1130. 10.3389/fmicb.2018.01130 29946299 PMC6005880

[B44] Siddaramappa S. Challacombe J. F. Delano S. F. Green L. D. Daligault H. Bruce D.. (2012). Complete genome sequence of *Dehalogenimonas lykanthroporepellens* type strain (BL-DC-9T) and comparison to *Dehalococcoides* strains. Stand. Gen. Sci. 6, 251–264. 10.4056/sigs.2806097 22768368 PMC3387798

[B45] Smidt H. Leest M. V. Oost J. V. D. De Vos W. M. (2000). Transcriptional regulation of the CPR gene cluster in ortho- chlorophenol-respiring desulfitobacterium dehalogenans. J. Bacteriol. 182, 5683–5691. 10.1128/JB.182.20.5683-5691.2000 11004165 PMC94688

[B46] Tang S. Edwards E. A. (2013). Identification of *Dehalobacter* reductive dehalogenases that catalyse dechlorination of chloroform, 1,1,1-trichloroethane and 1,1-dichloroethane. Philosoph. Trans. R. Soc. B Biol. Sci. 368,20120318. 10.1098/rstb.2012.0318 23479748 PMC3638459

[B47] Trinquier A. Durand S. Braun F. Condon C. (2020). Regulation of RNA processing and degradation in bacteria. Biochimica et Biophysica Acta (BBA) - Gene Regulatory Mechanisms 1863, 194505. 10.1016/j.bbagrm.2020.194505 32061882

[B48] Tsukagoshi N. Ezaki S. Uenaka T. Suzuki N. Kurane R. (2006). Isolation and transcriptional analysis of novel tetrachloroethene reductive dehalogenase gene from *Desulfitobacterium* sp. strain KBC1. Appl. Microbiol. Biotechnol. 69, 543–553. 10.1007/s00253-005-0022-x 16172885

[B49] Türkowsky D. Jehmlich N. Diekert G. Adrian L. von Bergen M. Goris T. (2018). An integrative overview of genomic, transcriptomic and proteomic analyses in organohalide respiration research. FEMS Microbiol. Ecol. 94, fiy013. 10.1093/femsec/fiy013 29390082

[B50] Wagner D. D. Hug L. A. Hatt J. K. Spitzmiller M. R. Padilla-Crespo E. Ritalahti K. M.. (2012). Genomic determinants of organohalide-respiration in *Geobacter lovleyi*, an unusual member of the Geobacteraceae. BMC Gen. 13, 200. 10.1186/1471-2164-13-200 22616984 PMC3403914

[B51] Wang P.-H. Tang S. Nemr K. Flick R. Yan J. Mahadevan R.. (2017). Refined experimental annotation reveals conserved corrinoid autotrophy in chloroform-respiring *Dehalobacter* isolates. ISME J. 11, 626–640. 10.1038/ismej.2016.158 27898054 PMC5322310

[B52] Wang S. Qiu L. Liu X. Xu G. Siegert M. Lu Q.. (2018). Electron transport chains in organohalide-respiring bacteria and bioremediation implications. Biotechnol. Adv. 36, 1194–1206. 10.1016/j.biotechadv.2018.03.018 29631017

[B53] Yan J. Bi M. Bourdon A. K. Farmer A. T. Wang P.-H. Molenda O.. (2018). Purinyl-cobamide is a native prosthetic group of reductive dehalogenases. Nat. Chem. Biol. 14, 8–14. 10.1038/nchembio.2512 29106396 PMC6081238

