# Supplementary material for: Stoichiometry of the Gene Products From the Tetrachloroethene Reductive Dehalogenase Operon pceABCT
Source: Front Microbiol. 2022 Feb 23;13:838026. doi: 10.3389/fmicb.2022.838026 (PMC8905343; doi:10.3389/fmicb.2022.838026)
Supplement: Supplementary file 1 [file Data_Sheet_1.PDF]

Supplementary Material

1 SUPPLEMENTARY DATA

1.1 *in-silico* RNA study revealed a hairpin structure in the *pceBC* region of *pce* operon in *D. restrictus*

In-silico analysis on the sequence of the *pce* operon revealed the presence of a possible hairpin-loop structure in the RNA of the *pceBC* intergenic region of *D. restrictus* and *D. hafniense* strain TCE1. In Supplementary Figure 1A, the genetic organization of the *pce* operon is shown and the star indicates the location of the predicted hairpin-loop structure in the *pceBC* intergenic region. Supplementary Figure 1B shows the structure of the predicted hairpin-loop structure.

1.2 Preliminary shotgun MS analysis on *D. restrictus*

Preliminary MS analyses were conducted on cell-free extracts of *D. restrictus*. This represented the first step in the design of the proteotypic peptides for the quantification of the Pce proteins. As showed in the Supplementary Figure 2, PceA and PceT resulted as highly detected with MS analysis where most the protein peptides could be detected. On the other hand, the detection of PceB and PceC proteins resulted in a much lower peptide coverage compared to PceA and PceT, likely due to their nature of integral membrane proteins.

1.3 Validation of PRM-based quantification of PceB

A validation experiment of the quantification of the PceB protein by PRM using a single targeted peptide (with amino acid sequence LANHPAK, Table 2) was performed by applying 480 fmol of the corresponding heavy-labelled peptide to three replicates of 856 fmol of the 43-aa long synthetic fragment of PceB (ProteoGenix, Schiltigheim, France) with the sequence: N_2-EYQAIGMGFIFFGGTALIPAIITYRLANHPAKKIRESSDTISA-COOH. The synthetic PceB fragment was initially dissolved in 50% DMSO and quantified by the Pierce BCA assay at 1.25 mg/mL. PRM analysis revealed an averaged light/heavy (L/H) ratio of 1.47, a recovery that was 19% off in comparison to the expected L/H ratio (856/480 = 1.78). This result was considered as acceptable considering the precision of the BCA assay and the PRM analytical variations. The mean analytical coefficient of variation (CV) of the LANHPAK heavy-labelled peptide was determined here by using five technical replicates and was found at 13.45%.

2 SUPPLEMENTARY TABLES AND FIGURES

2.1 Supplementary Tables

Supplementary Tables are provided in a separate Excel sheet.

Supplementary Table 1 presents the parameters and raw data of the quantitative PCR analysis.

Supplementary Table 2 shows the raw and analysed data from the PRM quantitative proteomics. The data from both biological replicates (A, B) are given.

Supplementary Table 3 displays a survey of proteomic studies applied to the Firmicutes OHRB.

2.2 Supplementary Figures

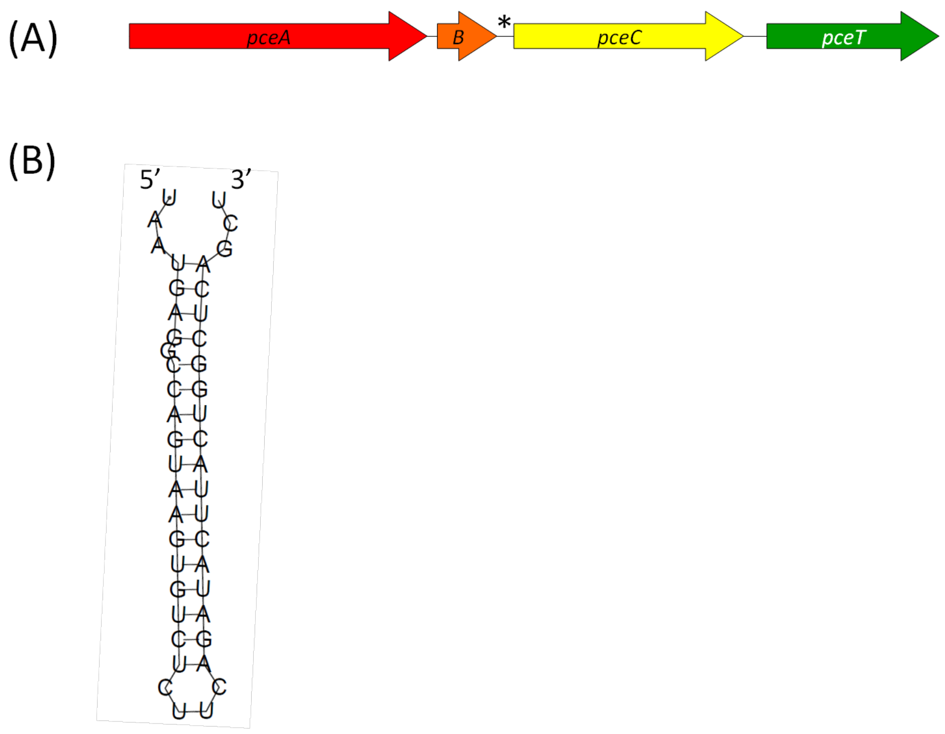

Figure S1. The *pceABCT* gene cluster of *D. restrictus*. **(A)** Genetic organization of the gene cluster. **(B)** Prediction of a putative hairpin loop structure in the *pceBC* intergenic region. The star indicates the position of the hairpin loop in the gene cluster.

PceA

```
  1 MGEINRRNFLKASMLGAAAAAVASASAVKGMVSPLVADAADIVAPITETSEFPYKVDAKYQRYNSLKNFFEKTFDPEANK
 81 TPIKFHYDDVSKITGKKDTGKDLPTLNAERLGIKGRPATHTETSILFHTQHLGAMLTQRHNETGWTGLDEALNAGAWAVE
161 FDYSGFNAAGGGPGSVIPLYPINPMTNEIANEPVMVPGLYNWDNIDVESVRQQGQQWKFESKEEASKILKKATRLLGADL
241 VGIAPYDERWTYSTWGRKILKPCKMPNGRTKYLPWDLPKMLSGGGVEVFGHAKFEPDWEKYAGFKPKSVIVFVLEEDYEA
321 IRTSPSVISSATVGKSYSNMAEVAYKIAVFLRKLGYYAAPCGNDTGISVPMAVQAGLGEAGRNGLLITQKFGPRHRIAKV
401 YTDLELAPDKPRKFGVREFCRLCKKCADACPAQAISHEKDPKVLQPEDCEVAENPYTEKWHLDSNRCGSFWAYNGSPCAN
481 CVAVCSWNKVETWNHDVARIATQIPLLQDAARKFDEWFGYNGPVNPDERLESGYVQNMVKDFWNNPESIKQ
```

PceB

```
  1 MNIYDVLIWMALGMTALLIQYGIWRYLKGKGKDTIPLQICGFLANFFFIFALAWGYSSFSEREYQAIGMGFIFFGGTALI
 81 PAIITYRLANHPAKKIRESSDTISA
```

PceC

```
  1 VKTKKNKAELENRRGWEYYYQFSLLLTAIIAILYGVFWAPQSVDYKGIIQKNVLGVISIEKMMGNQHAYKIDTAQGRFYA
 81 VCDSAIGYQSKVEAMTIVNEKGLIEKVIITKQGETPVFFERLTDQKYFDGFQGLAIKEPIYLGGAYGYSGYLGSIKTNNY
161 IDRVTGSTVSSHAVAEAVNKGNSYLSGQFFNTQWANPYDLFQLSWKDMAMIAMFLIAFASAFIKKLVKIRLAFLLVSVVV
241 LGFLVNQFVTGSLLLSAITLQIPRITNLKWYVLMAGSLGFIILLGKNLYCAWICPFGAVQEILNKAAGFKSLNISQKTIK
321 ILRLVAPTILWVALLLGTLLGDYGTLDYQPFGALFLFKSVWLMWLMLPIFLFMSLFISRFYCKFFCPVGFIYNLLNRWRN
401 EEVRIWKQRVDRLKRKKKEEQETWSSHS
```

PceT

```
  1 MKQFELGQYKGLNVKRFDTTVQEEEIQQALDYIIGSFDEIEEEKRNEPIKTNDYVIVDIDGYEKDATVPVIRNIDTKLIV
 81 GSEGVFREVSANLLGKKMGDTVTFESVIQPDALEFQRWWGSEFTFTVKIKSVFVVKKPELTEELIRKVEPDLKNLKDLKN
161 MLALKITHEKEGKEREANILLIFQALVKQCKYEFDEEELDSAAEDLYKKFTEELKIVDDMELMEYLIHRKITADQLLAEC
241 KEEASRRILWELMINSVIEKEEINLTPDEIKYLEKRINESRQNGQLPEEFMDINFLLASYLRKKTIDYLLKINLAS
```

ATP synthase

```
  1 MNLRPEEISSIIKQQIERYESALEVVDVGTVIQVGDGIARVYGLEKAMSGELLEFPGGIYGMAMNLEEDNIGCVILGPYA
 81 AIKEGDQVKRTGRIVEVPVGESLIGRVVNVLGQPVDGKGEIIPAGYRPIESDAPGVVDRKRVNEPLQTGLKSIDSMVPIG
161 RGQRELIIGDRQTGKTALAVDTIINQKGQNCICIYVAIGQKQSTVASVVKKLEEHGAMEYSIVVMATASDPAPMLYIAPY
241 SGCAIGEYFRDKGQHVLVVYDDLSKQAVAYRELSLLLKRPPGREAYPGDVFYLHSRLLERAAKLSPEKGSGSLTALPIIE
321 TQAGDVSAYIPTNVISITDGQIFLESDLFFSGFRPAINVGISVSRVGGDAQIKAMKQVAGQLRLDLAAYRELAAFAQFGS
401 DLDKATQARLNRGQKTMEILKQGQYQPMPVEEQIIVIFAATKGYIDDVPMERMKEFEQELLRFMRTTQIPEKIRTEKALS
481 NELMKEIGDAINEFKQGFLA
```

Figure S2. Graphical representation of sequence coverage of Pce proteins and F1 α-subunit of ATP synthase by shotgun MS analysis. The peptides that were detected via discovery MS analysis (i.e. sequence coverage) are shaded in grey. Red underlines indicate the peptides which were used in the PRM quantitative proteomic analysis.

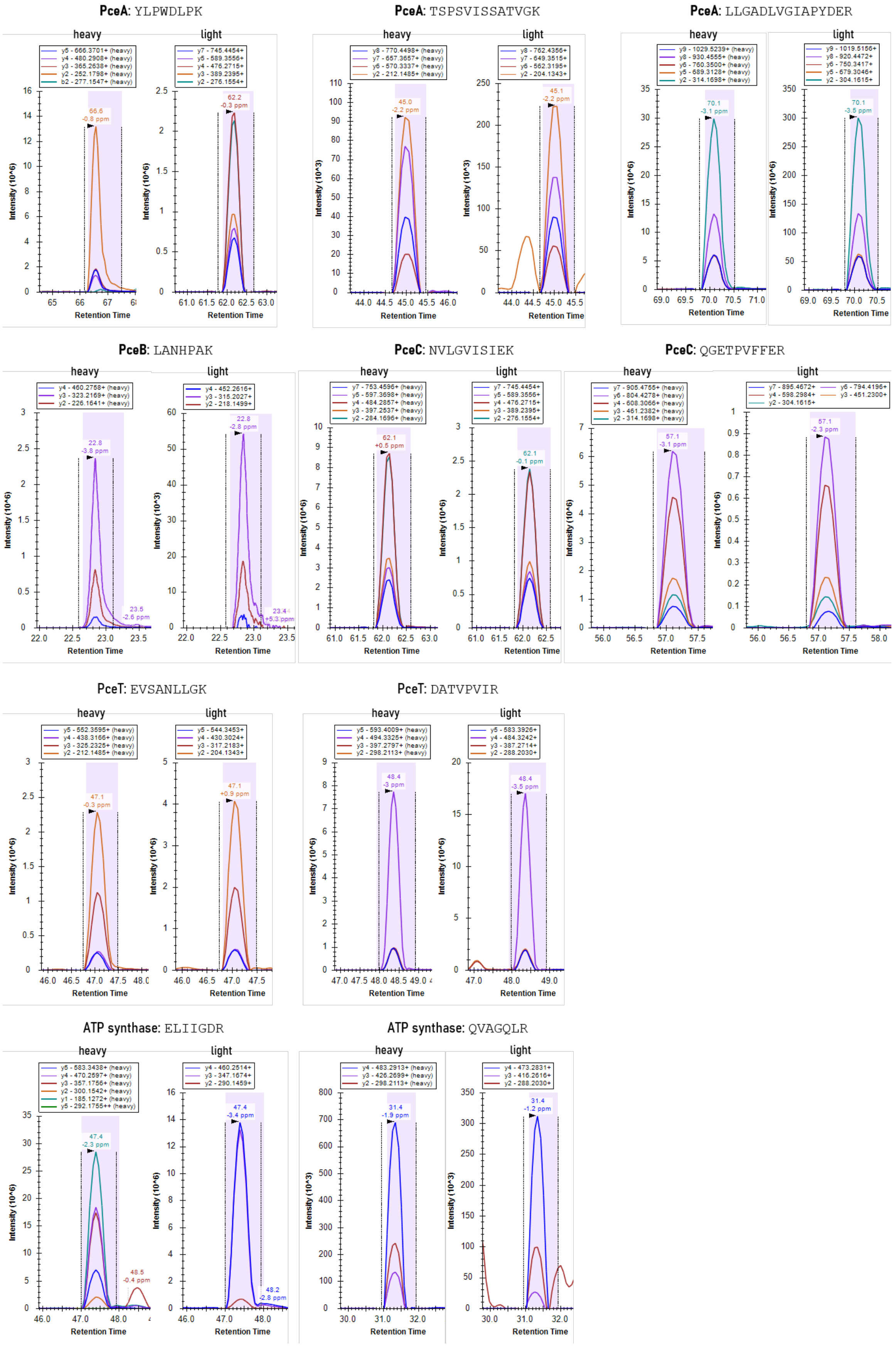

Figure S3. Representative examples of PRM spectra and transitions of all selected heavy-labelled peptides used in the quantification analysis.

(A)

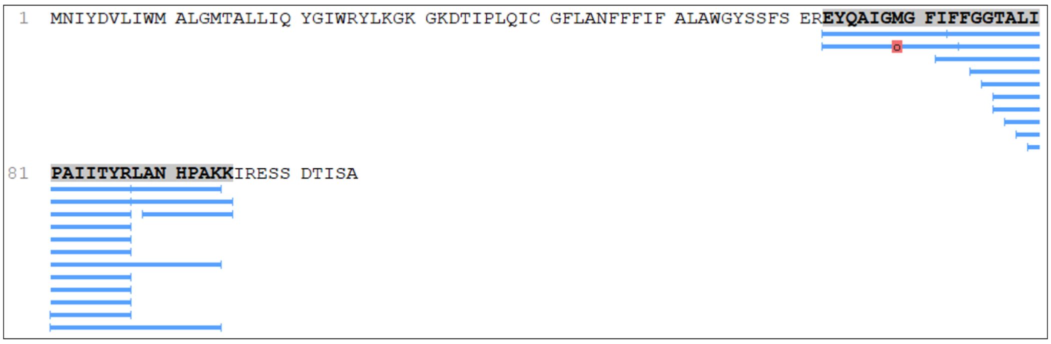

(B)

#	Peptide	Unique	-10lgP	Mass	Length	ppm	m/z	RT	Area Sample 1
1	I.PAIITYRLANHPAK.K	•	91.19	1563.8885	14	-0.4	782.9512	74.67	4.68E5
2	I.PAIITYR.L	•	80.85	832.4807	7	1.2	417.2481	78.08	1.12E8
3	G.TALIPAIITYR.L	•	74.61	1230.7335	11	-0.3	411.2516	71.79	9.67E7
4	F.GGTALIPAIITYR.L	•	73.37	1344.7765	13	-5.1	673.3921	71.92	4.98E7
5	G.GTALIPAIITYR.L	•	67.11	1287.7550	12	-2.4	644.8832	71.79	1.92E7
6	F.FGGTALIPAIITYR.L	•	66.05	1491.8448	14	-0.1	746.9296	74.29	1.54E7
7	L.IPAIITYR.L	•	63.26	945.5647	8	0.0	473.7896	71.01	1.33E7
8	I.FFGGTALIPAIITYR.L	•	63.23	1638.9133	15	-2.5	820.4619	76.64	9.82E6
9	F.IFFGGTALIPAIITYR.L	•	61.42	1751.9973	16	0.0	877.0059	78.25	1.22E6
10	G.TALIPAIITYRLANHPAK.K	•	61.22	1962.1414	18	1.2	491.5432	68.48	8.11E5
11	R.LANHPAK.K	•	57.40	749.4184	7	-7.5	375.7137	22.95	6.8E8
12	R.EYQAIGMGFI.F	•	56.76	1127.5321	10	0.2	564.7734	75.08	2.21E6
13	A.LIPAIITYR.L	•	55.67	1058.6488	9	-2.1	530.3306	66.00	1.8E7
14	L.ANHPAKK.I	•	55.23	764.4293	7	-10.3	383.2180	22.79	9.25E5
15	R.LANHPAKK.I	•	52.64	877.5134	8	3.2	439.7654	41.35	6.21E5
16	R.EYQAIGM(+15.99)GFIF.F	•	51.02	1290.5955	11	0.5	646.3053	76.33	5.89E6
17	T.ALIPAIITYR.L	•	50.34	1129.6859	10	3.0	377.5704	65.01	1.21E5

Figure S4. LC-MS/MS analysis of the PceB synthetic fragment. (A) Peptide coverage. (B) List of PceB peptides identified and their relative abundance (peak area) found. The peptide LANHPAK (11) represents a major cleavage product, whereas some minor, miss-cleaved forms such as LANHPAKK (15) could be still identified, which, however, represented less than 1% of the overall abundance of the major fully cleaved fragment.
